# Glyoxal-Lysine Dimer, an Advanced Glycation End Product, Induces Oxidative Damage and Inflammatory Response by Interacting with RAGE

**DOI:** 10.3390/antiox10091486

**Published:** 2021-09-17

**Authors:** Hee-Weon Lee, Min Ji Gu, Yoonsook Kim, Jee-Young Lee, Seungju Lee, In-Wook Choi, Sang Keun Ha

**Affiliations:** 1 Korea Food Research Institute, 245, Nongsaengmyeong-ro, Iseo-myeon, Wanju-gun 55365, Jeollabuk-do, Korea; 07989@kfri.re.kr (H.-W.L.); 50032@kfri.re.kr (M.J.G.); kimyus@kfri.re.kr (Y.K.); choiw@kfri.re.kr (I.-W.C.); 2 Molecular Design Team, New Drug Development Center, Daegu Gyeongbuk Medical Innovation Foundation (DGMIF), Daegu 41061, Korea; jyoung@dgmif.re.kr (J.-Y.L.); sjlee05@dgmif.re.kr (S.L.); 3 Division of Food Biotechnology, University of Science and Technology, Daejeon 34113, Korea

**Keywords:** glyoxal-lysine dimer, advanced glycation end product, receptor of advanced glycation end product, oxidative stress, NRF2/GLO-1, mitochondrial function

## Abstract

The glyoxal-lysine dimer (GOLD), which is a glyoxal (GO)-derived advanced glycation end product (AGE), is produced by the glycation reaction. In this study, we evaluated the effect of GOLD on the oxidative damage and inflammatory response in SV40 MES 13 mesangial cells. GOLD significantly increased the linkage with the V-type immunoglobulin domain of RAGE, a specific receptor of AGE. We found that GOLD treatment increased RAGE expression and reactive oxygen species (ROS) production in mesangial cells. GOLD remarkably regulated the protein and mRNA expression of nuclear factor erythroid 2-related factor 2 (NRF2) and glyoxalase 1 (GLO1). In addition, mitochondrial deterioration and inflammation occurred via GOLD-induced oxidative stress in mesangial cells. GOLD regulated the mitogen-activated protein kinase (MAPK) and the release of proinflammatory cytokines associated with the inflammatory mechanism of mesangial cells. Furthermore, oxidative stress and inflammatory responses triggered by GOLD were suppressed through RAGE inhibition using RAGE siRNA. These results demonstrate that the interaction of GOLD and RAGE plays an important role in the function of mesangial cells.

## 1. Introduction

Advanced glycation end products (AGEs) are stable proteins with post-translational modifications formed through spontaneous reactions with glucose and metabolite such as glyoxal and methylglyoxal. They are a large, heterogeneous group of compounds resulting from nonenzymatic Maillard reactions between reducing sugars and proteins, lipids, or nucleic acids, which can be produced both in vitro and in vivo [1,2]. AGEs contribute to the development of cancer and many chronic diseases, including diabetes and diabetic complications and neurodegenerative, cardiovascular, and kidney diseases [3,4,5]. AGEs, compounds formed by non-enzymatic reactions, can be obtained exogenously through food or formed endogenously in the body [6]. The effect of AGE consumption on the development of chronic diseases has remained inconclusive. Several systematic reviews of recent animal and clinical trials have shown that ingestion of AGEs may increase risk factors associated with chronic conditions, such as chronic renal disorder, oxidative stress, and a diabetic environment [7,8]. In addition, previous studies have shown the effect of an inflammatory response and oxidative stress after AGE consumption between healthy and diseased conditions. [9,10]. AGEs have a significant impact on the development and development of kidney damage.

Various AGEs contribute to the development of chronic kidney disease and cause diverse kidney failures. The production of AGEs further increases the concentration of AGEs associated with chemical and structural kidney changes, leading to nephropathy [11,12]. Besides, the formation and accumulation of AGEs initiate an even more serious pathogenic relationship with nephropathy in diabetes. The receptor for AGEs (RAGE) is a multi-ligand receptor of the immunoglobulin superfamily of cell surface molecules that act as receptors for a variety of molecules [13]. The participation of RAGE leads to the transmission of signals that cause cell dysfunction and tissue destruction. Indeed, AGE-induced kidney damage is more severe in patients with diabetes. Therefore, research related to the AGE receptor for AGE in renal dysfunction has focused on diabetes. However, besides the serious effect on the kidneys under diabetes, AGE–RAGE is implicated in kidney complications associated with obesity, inflammation, and high blood pressure [9,14,15].

AGE-specific receptors are expressed on various cells and affect the regulation of AGE uptake and removal as well as cell activation. As a key receptor for AGEs, RAGE is a multi-ligand receptor present in various organs that crucially influences the inflammatory process. It accumulates in various tissues due to oxidative stress induced by AGE accumulation. The generation of these signals through the combination of AGEs and RAGE causes various diseases and processes, such as kidney disease, cancer, diabetes, vascular disease, and aging [12,16,17]. Normally, the expression of RAGE is low in normal tissues but can be very high under pathological conditions, as mentioned earlier. Moreover, the combination of RAGE and RAGE ligands increases the expression of RAGE in various cells. This positive feedback loop may partially explain why RAGE–ligand interactions can trigger sustained activation of the RAGE downstream pathway [13,18]. Thus, the interaction between AGEs and RAGE is a key factor in the generation and exacerbation of lesions. Furthermore, the AGE–RAGE interaction triggers intracellular signaling that attenuate the nuclear factor erythroid 2-related factor 2 (NRF2) in a consistent cellular response. Besides, during this interaction, energy metabolism is affected via mitochondrial impairment [19].

Previous studies have shown that mitochondrial dysfunction via the AGE–RAGE axis is important for kidney lesion development. Oxidative stress-induced damage is the result of an imbalance between reactive oxygen species (ROS) production and detoxification and is directly linked to mitochondrial dysfunction. In addition, oxidative stress due to mitochondrial dysfunction affects kidney disease through excessive inflammatory reactions [11,20]. Several studies have shown that oxidative stress-triggered mitochondrial dysfunction occurs in inflammatory diseases, being key in kidney disease. Furthermore, the activity of the AGE–RAGE axis essentially affects the induction of ROS production in lesion development and progress [9]. However, the association between oxidative stress and AGE-mediated ROS as well as the mitochondrial dysfunction caused by this process remains unclear.

Abnormal ROS generation disrupts mitochondrial function and alters genetic mechanisms, triggering cellular damage. NRF2 is a key transcription factor that regulates genes involved in the antioxidant defense system. The expression of homeostasis regulators such as glyoxalase 1 (GLO1), which are regulated by NRF2, plays a role in preventing renal lesions from excessive inflammatory reactions through antioxidant reactions in mesangial cells [21]. NRF2 remains inactive while binding to Kelch-like ECH-associated protein 1 (KEAP1) in the cytoplasm [19]. Under an oxidative stress environment, the active site cysteine residue of KEAP1 is oxidized, preventing KEAP1 from interacting with NRF2. NRF2 then moves to the nucleus and activates the target gene through an antioxidant response element, which suppresses oxidative stress [22].

Excessive production of ROS and mitochondrial dysfunction play an important role in AGE–RAGE-mediated kidney disease. In addition, the oxidative stress reaction that occurs in this process is regulated by the NRF2/GLO1 signaling pathway [23,24]. The purpose of this study was to investigate the oxidative stress and inflammatory response by GOLD in mesangial cells. Our findings show that the interaction of GOLD and RAGE is related to oxidative stress and inflammatory responses in mesangial cells. We believe that these results will be helpful in the study of the effect of GOLD on kidney mesangial cells.

## 2. Materials and Methods

### 2.1. Chemical Reagents and Antibodies

Glyoxal-lysine dimer (GOLD) was purchased from IRIS Biotech GMBH (Marktredwitz, Germany). DMEM/Nutrient Mixture F-12 (DMEM/F12) and fetal bovine serum (FBS) were purchased from Lonza (Basel, Switzerland). Propidium iodide (PI), 4′,6-diamidino-2-phenylindole (DAPI), and 2′,7′-dichlorodihydrofluorescein diacetate (DCF-DA) were purchased from Sigma-Aldrich (St. Louis, MO, USA). The tetraethylbenzimidazolylcarbocyanine iodide (JC-1) mitochondrial membrane potential (MMP) assay kit was purchased from Abcam (Cambridge, UK). Antibodies against target molecules were obtained from Cell Signaling (Danvers, MA, USA), unless otherwise stated.

### 2.2. Cell Culture

The murine mesangial cell line SV40 MES 13 was purchased from ATCC (Rockville, MD, USA). SV40 MES 13 cells were cultured in DMEM/F12 supplemented with 2 mM L-glutamine, 100 IU/mL penicillin, 100 μg/mL streptomycin, 14 mM HEPES, and 5% heat-inactivated FBS. SV40 MES 13 cells were then incubated in a fully humidified incubator at 37 °C with 5% CO_2_. SV40 MES 13 cells were maintained in the culture medium at a concentration of 500 μM for 24 h.

### 2.3. Measurement of ROS

ROS production was quantified under fluorescence microscopy (ZEISS, Oberkochen, Germany) using a DCF-DA probe. In addition, we conducted an experiment using the antioxidant, *N*-Acetyl-L-cysteine (NAC). Mouse mesangial cells were incubated with 10 μM DCF-DA in the dark for 30 min at 37 °C, rinsed with phosphate-buffered saline (PBS), and counted using an ELISA plate reader (Molecular Devices, CA, USA). The following settings were used: Excitation, 488 nm and emission, 522 nm.

### 2.4. Docking Study

The GOLD-binding site in RAGE is the same as that of the methylglyoxal-lysine dimer (GOLD), as demonstrated in a previous article [25]. The binding model between GOLD and RAGE was predicted by docking simulation, and the final model was suggested using Molecular Mechanics energies combined with the Generalized Born and Surface Area (MM-GBSA) calculation. All calculations were performed using Glide and Prime modules of MAESTRO (Schrödinger LLC, New York, NY, USA) in a Linux environment with default parameters [26].

### 2.5. MitoTracker Staining and Confocal Laser Microscopy

MitoTracker Orange CMTMRos (Invitrogen, Carlsbad, CA, USA), a mitochondria-specific cationic fluorescent dye, was used to label the mitochondria. Transfected SV40 MES 13 cells were incubated with 200 nM MitoTracker for 1 h at 37 °C and washed four times with heated PBS. Cells were imaged using a confocal laser scanning microscope (ZEISS).

### 2.6. Immunofluorescence Assay

The expression of AGEs, RAGE, NRF2, and mitochondrial genes in GOLD-stimulated mesangial cells was determined under fluorescence microscopy. SV40 MES 13 cells were cultured on glass coverslips (22 mm in diameter) at a density of 2 × 10^4^ cells and then incubated with GOLD (500 μM) for 2 h. Mesangial cells were rinsed with PBS and fixed with 3.7% formaldehyde in PBS for 30 min at 20–22 °C. They were permeabilized with 0.2% Triton X-100 in PBS for 1 h. Thereafter, they were washed in PBS and incubated with antibodies against AGEs, RAGE, NRF2, GLO-1, DRP1, or MFN1 overnight at 4 °C. After washing with PBS, cells were incubated for 1 h with fluorescein isothiocyanate (FITC)-conjugated anti-rabbit IgG in 0.2% Triton X-100 in PBS. Cells were rinsed thoroughly, mounted with glycerol:PBS (4:1), and photographed using an LSM 900 fluorescence microscope (ZEISS).

### 2.7. RNA Extraction and Reverse Transcription-Polymerase Chain Reaction (RT-PCR)

The mRNA expressions of several genes were measured by qRT-PCR. After GOLD treatment, total RNA was isolated from cultured cells using Trizol (Sigma, St. Louis, MO, USA), according to the manufacturer’s instructions, and used for cDNA synthesis. qRT-PCR was performed using specific primers (Table 1). cDNA was amplified in 20 μL of a PCR reaction mixture (1 μL each of forward and reverse primers, 8 μL of cDNA synthesis solution in pure water, and 10 μL of SYBR Green Master Mix) in a quantitative real-time PCR system. Fluorescence was measured at each cycle.

### 2.8. Immunoblotting

SV40 MES 13 cells were seeded into 60 mm cell culture plates at a density of 2 × 10^6^ cells/well and incubated with GOLD (500 μM). After treatment, cultured cells were rinsed with PBS and suspended in a homogenized lysis buffer. The supernatant was collected after centrifugation for 5 min at 12,000× *g* and 4 °C. The protein concentration was determined using a DC protein assay kit (Bio-Rad, Hercules, CA, USA), with BSA as the standard. Whole-cell lysates were separated using 6–15% SDS-PAGE and transferred to a nitrocellulose membrane (Bio-Rad Laboratories, Hercules, CA, USA). Membranes were blocked with 5% skim milk in Tris-buffered saline containing 0.2% Tween 20 at 20–22 °C for 1 h and probed with the appropriate primary (1:500) and secondary (1:5000) antibodies. Blots were developed using an enhanced chemiluminescence kit. In each experiment, the density ratio represents the relative intensity of each band normalized to that of β-actin as a control. The following antibodies were used: Phospho-ERK (#9101), ERK (#9102), phospho-JNK (#9258), JNK (#9251), phospho-p38 (#4551), p38 (#8690), and NRF2 (#12721), all from Cell Signaling Technologies (Danvers, MA, USA); TNF-α (sc12744), IL-6 (sc32296), IL-1β (sc12742), OPA1 (sc393296), Mfn1 (sc16664), and Drp1 (sc271583), all from Santa Cruz Biotechnology, (Dallas, TX, USA), RAGE (ab216329); IL-18 (ab74495), p21 (ab188224), p27 (ab32034), CDK2 (ab32147), and GLO-1 (ab171121), all from abcam (Cambridge, UK); and AGEs (bs1158R), all from BIOSS (Woburn, MA, USA).

### 2.9. RNA Interference

SV40 MES13 cells were plated into 6-well culture plates and transiently transfected with RAGE small interfering RNA (siRNA) (Santa Cruz, CA, USA) mixed with an siRNA transfection reagent (Invitrogen Biotechnology, Carlsbad, CA, USA), according to the manufacturer’s instructions. To knockdown endogenous RAGE, cells were transiently transfected with siRNA at a concentration of 50 nM for 20 h.

### 2.10. Assessment of Mitochondrial Transmembrane Potential (ΔΨm), Biogenesis, AGEs, and ATP Production

Mitochondrial membrane potential (MMP) was measured by staining mesangial cells with JC-1, a cationic dye that accumulates in energized mitochondria. The JC-1 solution (20 µM) was added to each dish 30 min prior to the end of the treatment, and then cells were rinsed with dilution buffer. Fluorescence images were captured under an LSM 900 fluorescence microscope.

Cells were fixed with fixing solution and stimulated with quenching buffer for mitobiogenesis at room temperature. The cultured cells were incubated with blocking buffer at 37 °C. After reacting the prepared primary antibody in all wells, the secondary antibody was incubated at room temperature. Cells had HRP One-Step Substrate Reagent added to each well, they were incubated at room temperature, and checked by adding a stop solution.

Total AGEs levels were investigated using enzyme-linked immunosorbent assay (ELISA) kits (Cell Biolabs, Inc., Beverly, MA, USA) according to the manufacturer’s instructions, and the results were normalized to total protein concentrations. The total AGE amount was measured using a microplate reader at 450 nm.

SV40 MES 13 cells were seeded into 96-well black microplates at a density of 2 × 10^4^ cells/well for ATP production. The culture medium was removed, the ATP incubation medium (KCL 15 mM, Tris–HCl 25 mM, EDTA 0.2 mM, KH_2_PO_4_ 1 mM, pH 7.4, 1% albumin, ADP 1 mM) and malate 100 mM was added, and the cells were incubated at 37 °C for 2 h. After the incubation medium was withdrawn and cells were washed with PBS, the incubation medium without ADP was added, and the cells were detached with a cell scraper. The cell supernatant was collected in a micro test tube, boiled with Tris–EDTA, and centrifuged at 1000× *g* at room temperature for 1 min. The supernatant was then collected for protein and ATP assay. Samples were placed in a 96-well plate (Corning Inc., Corning, NY, USA), and BSA protein (Sigma, St. Louis, MO, USA) with a series concentration (0–0.2 mg/mL) was utilized as the standard to normalize the assay. Optical absorption at 595 nm was measured. The quantity of ATP was measured using an ATP determination kit (A-22066, Eugene, OR, USA) following the manufacturer’s instructions. The reagents and reaction mixture were combined according to the protocol by molecular probes.

### 2.11. Flow Cytometry

Cell cycle progression was determined using flow cytometry. Mesangial cells were incubated with GOLD for 2 h, collected, and fixed with 4% formaldehyde at 4 °C overnight. After removal of the fixing buffer, cells were stained with 50 μg/mL PI solution containing RNase A (10 μg/mL) for 1 h in the dark. Fluorescence-activated cell sorting analysis was conducted on a CytoFLEX S Flow Cytometer, and data analysis was performed using Kaluza Analysis Software (Beckman Coulter, Indianapolis, IN, USA).

### 2.12. Statistical Analyses

For each experiment, data were obtained in triplicate and reported as the mean ± SEM. Comparisons of means between GOLD-treated cells and untreated control cells were conducted using analysis of variance and Student’s *t*-test. Significant differences were considered at *p* < 0.05.

## 3. Results

### 3.1. Effects of GOLD on AGEs and RAGE Levels

**Figure 1:**
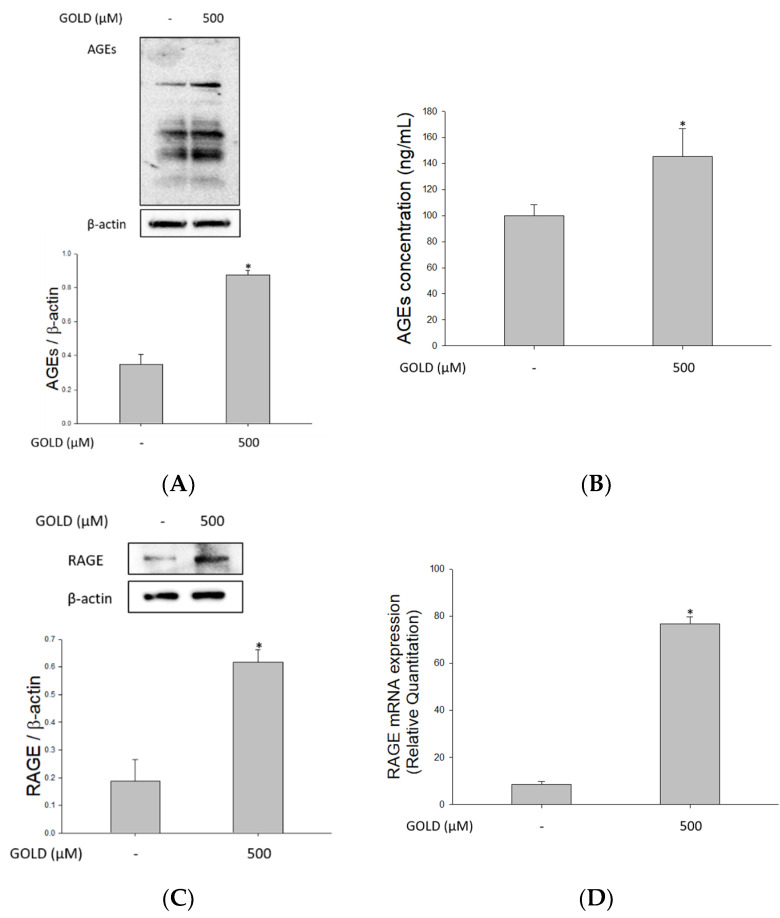

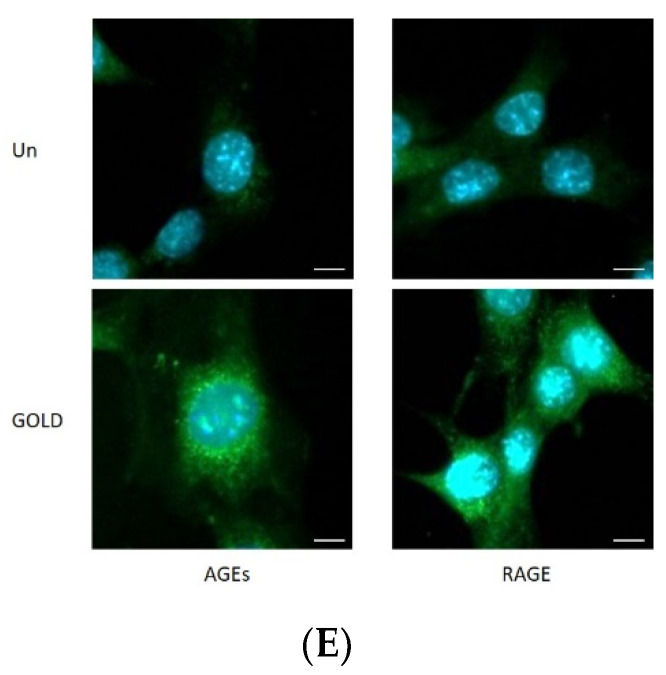
Effects of advanced glycation end products (AGEs) and expression of receptor for AGEs (RAGE) levels. SV40 MES 13 cells were stimulated with the glyoxal-lysine dimer (GOLD; 500 μM) for 24 h. (**A**,**C**) The protein levels of AGEs and RAGE were determined using Western blotting, with β-actin as an internal control. (**B**) AGE production was measured using an ELISA kit (CELL BIOLABS, STA-817). (**D**) RAGE expression was determined using RT-qPCR. GAPDH was used as an internal control. (**E**) SV40 MES 13 cells were incubated with a vehicle (medium) or GOLD for 24 h. After stimulation, cells were treated with anti-AGE and -RAGE primary antibodies, followed by fluorescein isothiocyanate (FITC)-labeled anti-rabbit IgG. Cells were detected using fluorescence microscopy at ×400 magnification. Scale bar, 20 μm. Data are expressed as the mean ± SEM of three independent experiments. * *p* < 0.05 indicates a significant difference compared to the untreated group.

AGE accumulation in the body can cause several serious disorders. Currently, RAGE is considered a key factor that transmits AGE signals. Various lesions are known to be exacerbated through several signaling processes via RAGE. Therefore, we determined the formation of AGEs and RAGE expression in the presence of GOLD. Besides, we investigated the association between AGE formation and RAGE distribution in mesangial cells. As shown in Figure 1A,B, when GOLD was added, AGEs were detected not only intracellularly but also extracellularly. We confirmed that the protein and mRNA levels of RAGE increased with AGE production (Figure 1C,D). In addition, immunofluorescence analysis revealed that GOLD induced the expression of RAGE and its translocation to the cell membrane in SV40 MES 13 cells (Figure 1E). Collectively, these findings demonstrate that GOLD induces AGE levels and RAGE expression compared with untreated cells.

### 3.2. Prediction of Binding Model between GOLD and RAGE

**Figure 2:**
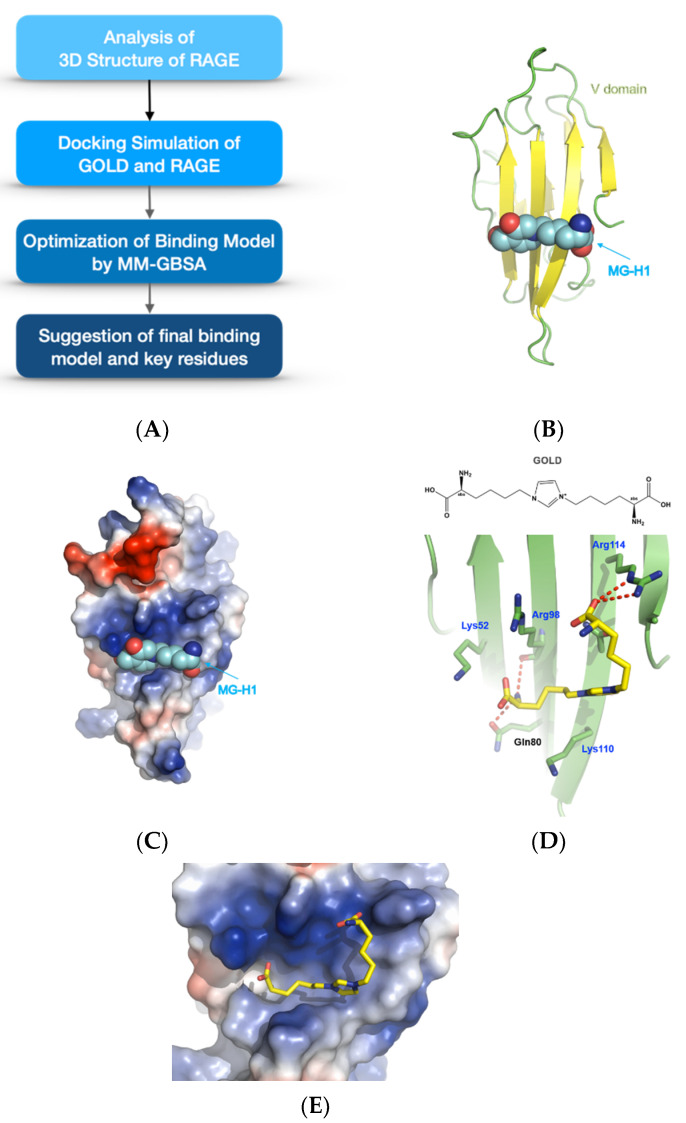
Prediction of the binding model between the glyoxal-lysine dimer (GOLD) and RAGE. Signaling in the mechanisms of GOLD treatment was investigated. Mesangial cells were stimulated with GOLD (500 μM) for 24 h. (**A**) Steps of the docking simulation to determine the binding model of GOLD and RAGE (**B**) Binding structure of the V domain of RAGE and the known ligand methylglyoxal-derived hydroimidazolone-1 (MG-H1) according to nuclear magnetic resonance (NMR) (2MOV.pdb). (**C**) Electrostatic surface model of the V domain of RAGE. (**D**) Binding model of GOLD and the V domain of RAGE according to the docking simulation. The red dashed line represents the H-bond. (**E**) Electrostatic surface model of GOLD and RAGE. Blue indicates the positively charged region, and red indicates the negatively charged region. There are many positively charged residues (colored blue) around the GOLD-binding site.

The AGE–RAGE axis is a key mechanism in kidney disorders. To examine whether the stimulatory effect of GOLD on this receptor is related to the structural interaction as well as RAGE expression, the structural binding between GOLD and RAGE was evaluated in mesangial cells. It is known that several RAGE ligands, such as methylglyoxal-derived hydroimidazolone-1 (MG-H1) and GOLD, bind to the V domain of RAGE [25,27,28]. The binding site of AGEs in RAGE comprises several positively charged residues, such as Lys and Arg, and these residues formed an H-bond interaction with GOLD (Figure 2B,C). The final binding model of GOLD and RAGE is depicted in Figure 2D,E. The amine group of GOLD participated in the hydrogen bond with the backbone carbonyl group of Arg98 and side chain carbonyl of Gln80. The carboxyl acid of the imidazole ring formed an H-bond with Arg114. The key feature of the binding of GOLD to RAGE is the H-bond interactions with amino acid residues. An H-bond interaction is a major feature that determines the specificity of ligand binding to the target protein [29]. It was confirmed that the Lys moiety of GOLD contributes to its stable binding by forming hydrogen bonds with amino acid residues of RAGE.

### 3.3. Effects of GOLD on Inflammatory Mediators, ROS Production, and Cell Cycle

**Figure 3:**
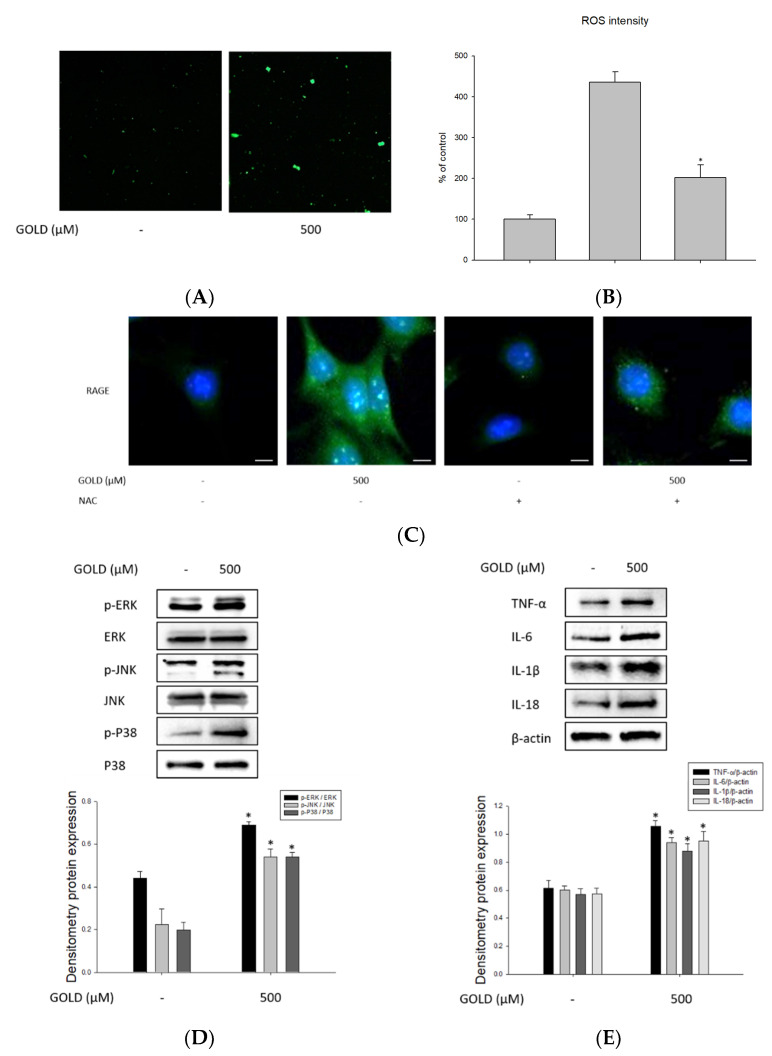

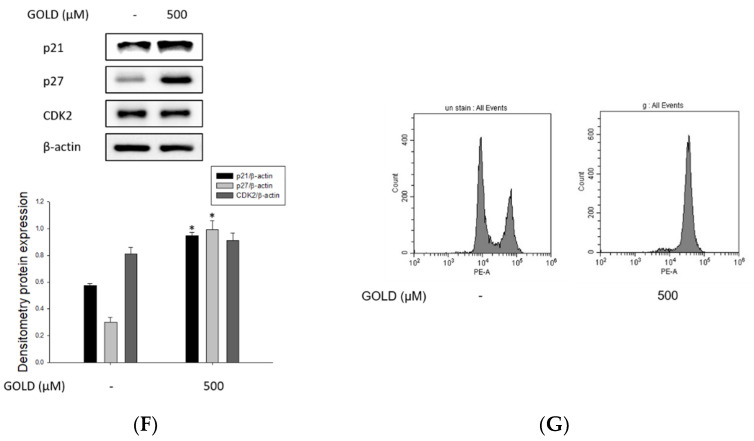
Effects of GOLD on inflammatory mediators, ROS production, and cell cycle. SV40 MES 13 cells were treated with GOLD (500 μM) for 30 min or 24 h. (**A**,**B**) Reactive oxygen species (ROS) production was determined as described in the Materials and Methods. Mesangial cells were treated with vehicle (medium) in the presence or absence of GOLD (500 μM) for 30 min. The amount of ROS in mesangial cells was analyzed using confocal microscopy. (**C**) Cells were incubated with anti-RAGE primary antibodies, followed by FITC-labeled anti-rabbit IgG. Cells were observed under fluorescence microscopy at ×400 magnification. (**D**,**E**) The phosphorylation levels of MAPK and the production of proinflammatory cytokines were detected using Western blotting. (**F**) Mesangial cells were incubated for 30 min in the absence or presence of GOLD (500 μM). The levels of cell cycle-related proteins were evaluated using Western blotting. (**G**) SV40 MES 13 cells were cultured in a medium containing GOLD (500 μM) for 24 h, fixed with 70% ethanol, stained with propidium iodide solution, and assessed using fluorescence-activated cell sorting. β-actin levels were used as the internal control. Scale bars, 20 μm. Data are expressed as the mean ± SEM of three independent experiments. * *p* < 0.05 indicates a significant difference compared to the untreated group.

It is well known that the AGE–RAGE axis causes excessive ROS production of cells in many diseases. In addition, these ROS are closely related to cell damage through oxidative stress. As shown in Figure 3A,B, we determined the effect of GOLD on ROS production using an ELISA reader and confocal microscopy. Treatment with 500 μM GOLD increased ROS production by approximately 3.7-fold compared with that in the untreated group. We used the antioxidant NAC to determine the effect of ROS. Treatment with NAC reduced the production of ROS by more than 50%. This indicates that the binding of GOLD to RAGE is associated with inflammatory responses through ROS production in mesangial cells. ROS is key to oxidative stress and is an important component of the signaling system through direct interaction with RAGE. Therefore, we performed fluorescence staining to investigate the relationship between the AGE–RAGE axis and ROS production by GOLD stimulation, which is important for the oxidative stress response. Immunofluorescence analysis revealed that the distribution of RAGE, according to AGE stimulation, and ROS generated along the AGE–RAGE axis interact in mesangial cells (Figure 3C).

Various factors are involved in the inflammatory response in kidney diseases. Therefore, we examined whether the activating effect of GOLD on the inflammatory response is MAPK pathway dependent. Our results showed that GOLD dramatically increased the phosphorylation of MAPK proteins, extracellular signal-regulated kinase 1/2 (ERK1/2), c-Jun N-terminal kinases (JNK), and p38 compared to that in the untreated group (Figure 3D). Furthermore, we investigated the expression of proinflammatory cytokines, such as tumor necrosis factor-α (TNF-α), interleukin (IL)-6, IL-1β, and IL-18. The production of proinflammatory cytokines was remarkably induced by incubation with GOLD (Figure 3E). These results indicate that the GOLD-induced inflammatory responses are related to the MAPK and inflammatory cytokine signaling pathways.

Next, to assess the cell cycle and identify cell cycle regulators that modulate mesangial cell differentiation, we first performed flow cytometry and Western blot analyses to determine changes in cell proliferation induced by GOLD. Flow cytometry analysis showed that GOLD significantly regulated the cell cycle (Figure 3F,G). Therefore, we further investigated the expression of cell cycle-related genes, such as p21, p27, and CDK2. The expression of p21 and p27 was upregulated by GOLD treatment, but levels of CDK2 were unchanged in SV40 MES 13 cells. Collectively, these results indicate that GOLD stimulates inflammatory responses and oxidative stress in SV40 MES 13 cells. In addition, these processes affect cell cycle regulation and cell differentiation by regulating cell cycle-related factors.

### 3.4. Effects of GOLD on NRF2 and the GLO1 Signaling Pathway

**Figure 4:**
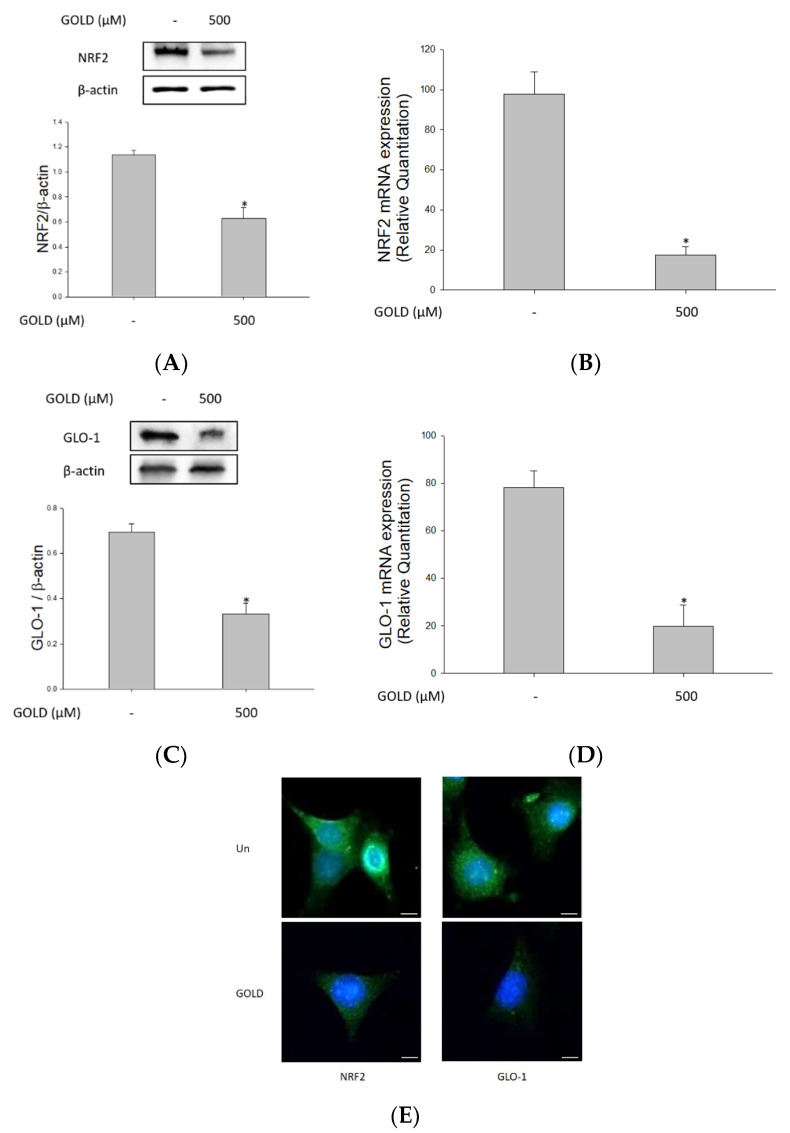
Effects of GOLD on nuclear factor erythroid 2-related factor 2 (NRF2) and the glyoxalase 1 (GLO1) signaling pathway. SV40 MES 13 cells were incubated in the presence or absence of GOLD (500 μM) for 24 h. (**A**,**C**) Protein levels of NRF2 and GLO1 were measured using Western blotting. (**B**,**D**) mRNA levels of NRF2 and GLO1 were determined using RT-qPCR. (**E**) Cells were incubated with anti-NRF2 and -GLO1 primary antibodies, followed by FITC-labeled anti-rabbit IgG. Cells were observed under fluorescence microscopy at ×400 magnification. β-actin levels were used as the internal control. Scale bars, 20 μm. Data are expressed as the mean ± SEM of three independent experiments. * *p* < 0.05 indicates a significant difference compared to the untreated group.

Next, we investigated the effect of GOLD on the expression of NRF2 and GLO1 in mesangial cells. The NRF2/GLO1 signaling system is an important antioxidant activity signaling system [21,30]. As shown in Figure 4A,C, GOLD significantly inhibited the expression of NRF2 and GLO1 in mesangial cells. Treatment of cells with GOLD resulted in decreased NRF2 and GLO1 mRNA levels compared to those in untreated cells (Figure 4B,D). When NRF2 is activated, it moves into the nucleus. Immunofluorescence microscopy confirmed the expression of NRF2 and revealed that translocation of NRF2 from the cytosol to the nucleus was suppressed by GOLD in SV40 MES 13 cells (Figure 4E). These results indicate that GOLD decreases the expression levels of NRF2 and GLO1.

### 3.5. Regulation of Mitochondrial Function by GOLD

**Figure 5:**
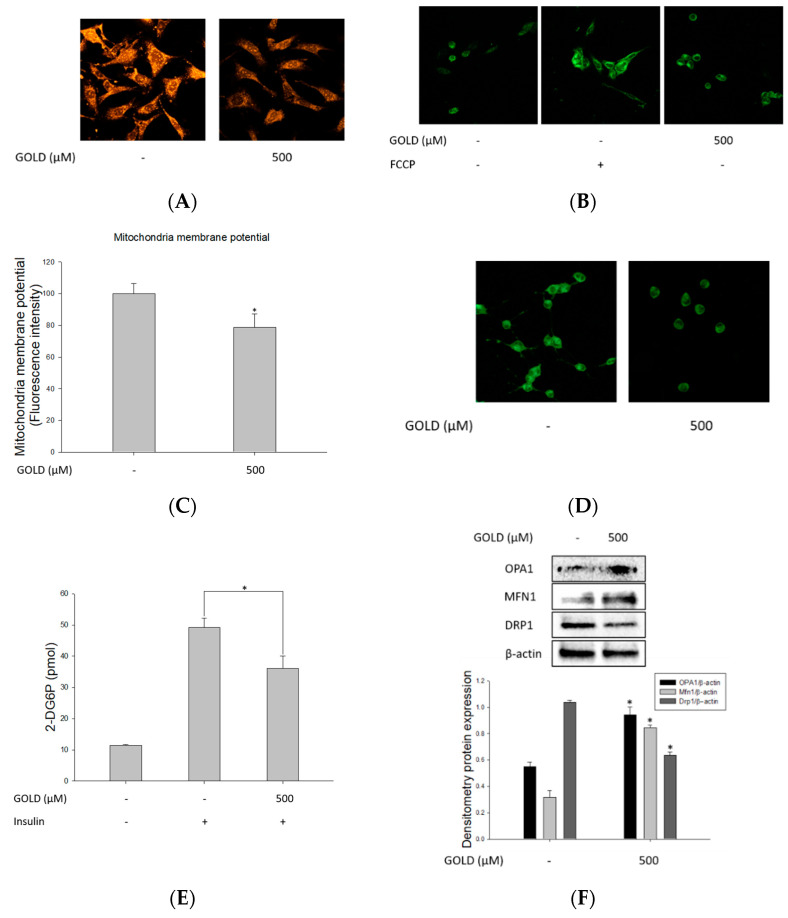

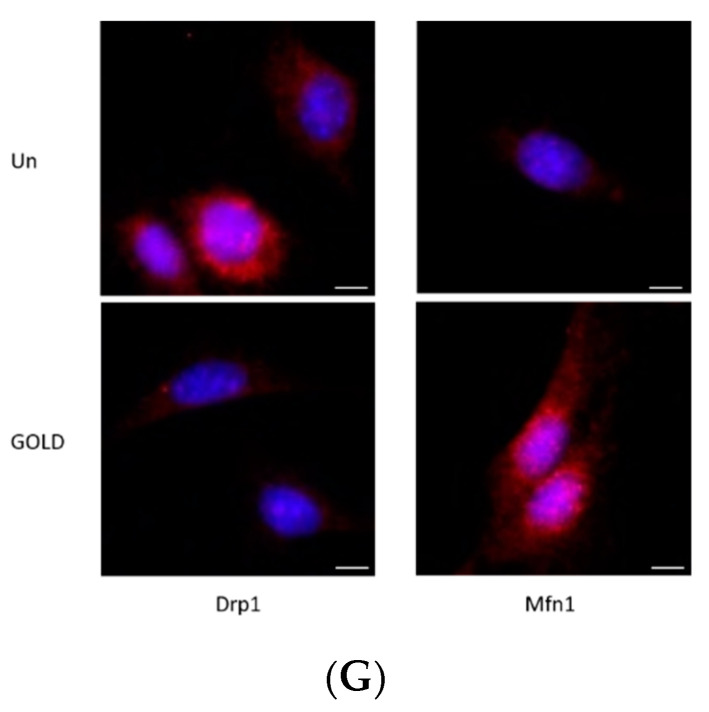
Regulation of mitochondrial function by GOLD. The expression and function of mitochondria under GOLD treatment (500 μM) were investigated. (**A**) SV40 MES 13 cells were incubated with MitoTracker Orange. Mitochondrial morphology and expression were investigated using confocal microscopy. (**B**) Mitochondrial membrane potential was measured using an ELISA kit. Mitochondrial genes were assessed in SV40 MES 13 cells. FCCP expression was measured as a positive control. (**C**) The mitochondrial membrane potential was determined using confocal microscopy and an ELISA kit. Quantitative analysis of tetraethylbenzimidazolylcarbocyanine iodide (JC-1)-stained cells using flow cytometry. SV40 MES 13 cells were pretreated with 500 μM GOLD for 24 h and then stained with JC-1 for 20 min. (**D**) Adenosine triphosphate (ATP) content in the untreated and GOLD-treated groups. Data are expressed as the mean ± standard deviation (represented by vertical bars; *n* = 3). (**E**) Basal glucose transport and insulin-stimulated glucose transport rates are shown. The basal rate refers to the rate of glucose transport in the absence of insulin. All values were normalized to the basal rate. (**F**) The expression of mitochondrial fusion and fission-related genes was investigated to determine mitochondrial dynamics using Western blotting. β-actin expression was measured as an internal control. (**G**) Cells were incubated with anti-DRP1 and –MFN1 primary antibodies, followed by Alexa 488-labeled anti-mouse IgG. Cells were observed under fluorescence microscopy at ×400 magnification. Data are expressed as the mean ± SEM of three independent experiments. * *p* < 0.05 indicates a significant difference compared to the untreated group.

To evaluate mitochondrial function triggered in GOLD-stimulated cells, we investigated the morphological and functional changes in mitochondria in SV40 MES 13 cells. First, we measured the accumulation and mass of mitochondria using MitoTracker under confocal microscopy. GOLD-stimulated cells showed decreased mitochondria expression and morphology compared to the control group (Figure 5A). As shown in Figure 5B,C, we further examined the JC-1 staining of GOLD-stimulated mitochondria to confirm mitochondrial membrane potential. These results show that MMP is depolarized by GOLD treatment in mesangial cells. Measurement of the MMP, as an indicator of mitochondrial activity, revealed a marked reduction in that of the GOLD-treated group, which was consistent with the changes in mitochondrial accumulation and morphology. Additionally, we identified cellular adenosine triphosphate (ATP) production as an essential function of the mitochondria, showing that GOLD suppressed ATP production in mesangial cells (Figure 5D). Moreover, we examined the effect of GOLD on glucose uptake during mitochondrial dysfunction. Treatment with GOLD significantly reduced the uptake of glucose in mesangial cells compared to that in the control group (Figure 5E).

Next, we determined the expression of mitochondrial fusion and fission-related genes to investigate mitochondrial dynamics. As shown in Figure 5F,G, the expression of the mitochondrial fission-related genes dynamin-related protein 1 (DRP1) and mitochondrial fission factor (MFF) was dramatically increased after incubation with GOLD, whereas that of mitofusin-1 (MFN1) and optic atrophy 1 (OPA1) was decreased in mesangial cells. Furthermore, we confirmed the changes in mitochondrial fission and fusion-related gene expression in GOLD-stimulated cells using confocal microscopy. These results suggest that GOLD reduces mitochondrial function by causing genetic and morphological changes in the mitochondria.

### 3.6. Changes in Signaling through RAGE Suppression

**Figure 6:**
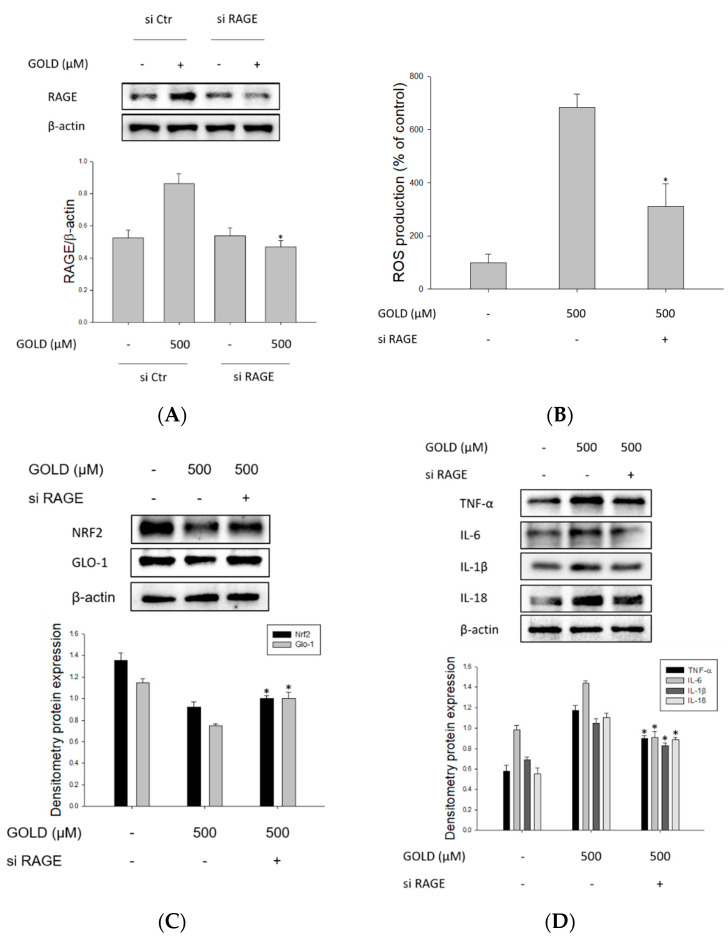

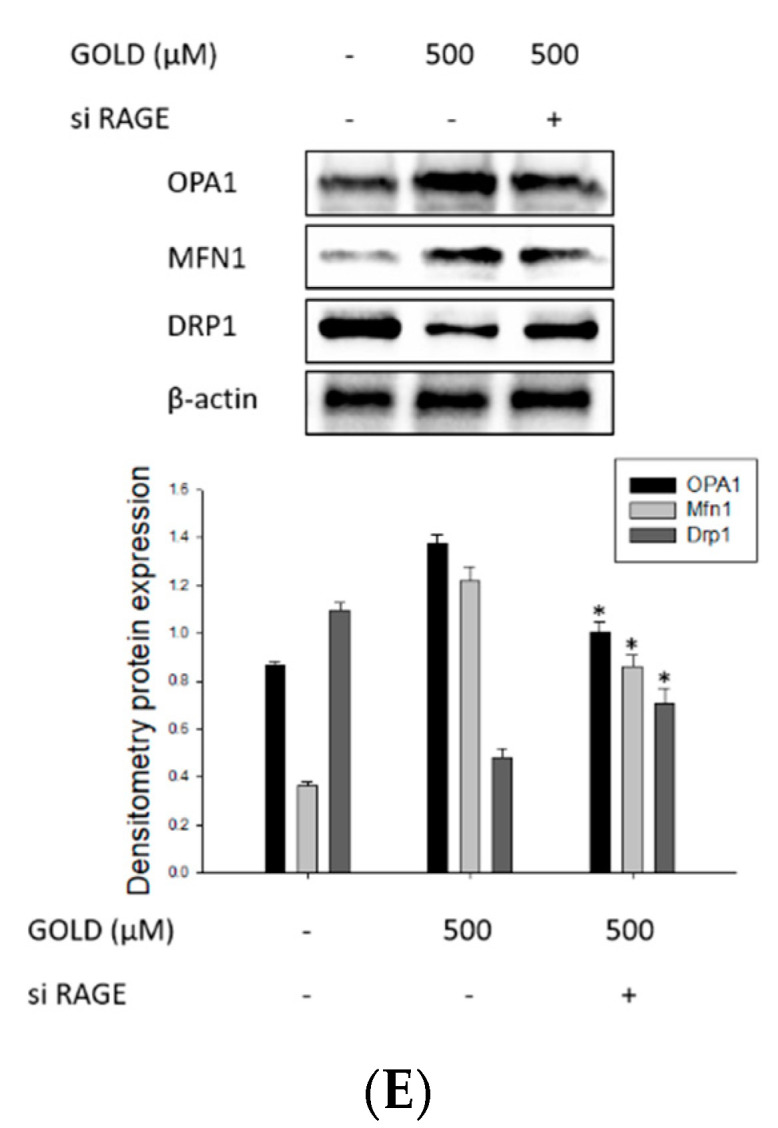
Changes in signaling through RAGE suppression. Effects of RAGE on GOLD-stimulated SV40 MES 13 cells. (**A**) SV40 MES 13 cells were transfected with control small interfering RNA (siRNA) or RAGE siRNA and then treated with GOLD. (**B**) SV40 MES 13 cells were transfected with RAGE siRNA and then stimulated with GOLD (500 μM) for 30 min. ROS levels were determined using confocal microscopy. (**C**,**D**) Protein levels of NRF2, GLO1, and proinflammatory cytokines were determined using Western blotting, with β-actin as an internal control. (**E**) Mitobiogenesis was measured by detecting mitochondrial genes in SV40 MES 13 cells. Data are expressed as the mean ± SEM of three independent experiments. * *p* < 0.05 indicates a significant difference compared to the GOLD-treated group.

Next, we examined whether RAGE signaling pathways contribute to inflammatory mediators and mitochondrial function in GOLD-activated mesangial cells and investigated changes in the inflammatory response by transfecting cells with RAGE siRNA. Prior to these experiments, we confirmed the knockdown efficiency of RAGE siRNA. siRNA reduced the expression of RAGE by approximately 46% (Figure 6A). As shown in Figure 6B, when RAGE was suppressed by siRNA, ROS production significantly decreased compared to that in the control siRNA transfection group. The NRF2/GLO1 signaling pathway was found to be closely related to the oxidative stress response caused by AGEs in numerous disorders. As expected, RAGE suppression increased the activity of the NRF2/GLO1 signaling pathway (Figure 6C). In kidney disease, inflammatory cytokine overproduction through the AGE–RAGE axis promotes lesion exacerbation. As shown in Figure 6D, it was confirmed that cytokine production induced by GOLD was inhibited through RAGE suppression. Stimulation with AGEs crucially induces RAGE activation, stimulating inflammatory signaling. We confirmed mitochondrial dynamics that mitochondrial function was also restored through the suppression of RAGE (Figure 6E). Taken together, these results suggest that the inflammatory response stimulates the overproduction of RAGE and inflammatory mediators, causing impaired kidney function. Furthermore, it was shown that by inhibiting mitochondrial function, these processes aggravate the mesangial function condition of mesangial cells.

## 4. Discussion

This study identified a specific mechanism through the effect that GOLD, a GO-induced AGE produced by glycation, produces excessive ROS production and induces an inflammatory response in mesangial cells. It also affects oxidative stress by changing the NRF/GLO1 signaling pathway in mesangial damage. We reported that GOLD increases RAGE expression on the surface of mesangial cells and causes mesangial damage by bonding to the surface of RAGE in silico. In many studies, the toxic effects of AGEs have been investigated, and related research is being conducted on various diseases. However, most studies have focused on the toxicity and disease induction caused by AGEs. In addition, most research is limited to AGEs such as pentosidine, N^∊^-(Carboxymethyl)lysine (CML), and MG-H1 [31,32,33]. Specific AGEs, such as the well-known CML and Nε-(Carboxyethyl)lysine (CEL), are harmful to the kidneys [34]. In this study, we have shown directly how GOLD itself has a detrimental effect in mesangial cells. We sought to determine the effect of GOLD on several important renal cells such as mesangial cells, epithelial cells, and podocytes based on AGE-related studies [35,36]. When we checked the effect of AGEs using various types of kidney cells, it was confirmed that the critical effect was shown on mesangial cells. Besides, we conducted a more comprehensive study that confirmed the inflammatory effect of GOLD and the oxidative stress mechanism by which the GOLD effect occurs in mesangial cells. Therefore, the study of GOLD could be crucial due to its effect on the damage of mesangial cells. Furthermore, the association of GOLD with mesangial cells could be important to unveil its association with other diseases. In addition, many previous studies have investigated the involvement of AGEs as well as GOLD in a variety of diseases and found they are associated with chronic kidney disease. Several studies have also investigated the toxicity of GO-derived AGEs [37,38]. These studies show that mesangial cell damage is increased through the regulation of RAGE expression and specific signaling by the AGE–RAGE axis. We demonstrated the interaction between downstream mechanisms through the signaling of GO-derived AGEs through the interaction of GOLD and RAGE in mesangial cells.

AGEs can be produced in various forms from several types of precursors that cause the Maillard reaction. The main factor that can trigger this reaction in our body is food intake [39]. Endogenous generation in living organisms, including humans, is also a critical factor in the formation of AGEs. The latter is the product of Maillard reactions initiated by a nonenzymatic reaction between a carbonyl compound and an amine group [40]. The reaction forms a number of Maillard reaction product intermediates with different molecular sizes and compositions depending on the reaction compound, and compounds with various compositions and molecular weights are involved in the formation of AGEs as they contain various peptides [41,42]. In particular, GOLD is a GO-derived AGE often produced by fructose. These dicarbonyl-derived AGEs represent major chemical modifications that accumulate in tissue proteins with age and in chronic diseases, such as diabetes and chronic kidney disease [34,43]. Therefore, glyoxal-derived AGEs have been investigated in various ways, some of which have been found to be directly involved in AGE formation and RAGE interaction [44,45]. GOLD is an AGE that shows strong toxicity in the body and is used as an important indicator of protein glycation [46]. In addition, the cross-linking of proteins, including Lys, glycosylated Lys, and Arg, rouses great interest because it significantly impairs physiological functions and induces various AGE-related diseases [47]. In particular, GOLD, which is produced from two Lys residues and glyoxal, was found in tissue as an important indicator of diseases related to glycation products. Despite the great advances made by these biochemical studies, data on how closely the glycation reaction of human proteins can be reproduced by model systems using amino acid–sugar mixtures remain limited.

The body produces and accumulates AGEs through various reactions from food. In this regard, many studies have focused on the effect of AGEs and found that AGEs are associated with various diseases in humans. AGEs are associated with the incidence of kidney disease and lesion exacerbation [4,8]. Although these studies have shown detrimental effects of AGEs, few have investigated the precise mechanisms triggering kidney damage. Most of the research is limited to studies on specific AGEs, such as CML and CEL. Herein, we have confirmed that GOLD induces damage to the mesangial and demonstrated the mechanism of mesangial damage in mesangial cells. Therefore, our study focuses on new goals in the treatment of kidney disease by discovering novel mechanisms by which AGE contributes to kidney damage.

RAGE, present on the surface of the plasma membrane, is a member of the Ig superfamily. It binds to AGEs on the cell surface and transmits an intracellular signal [18,48]. Therefore, it is important to determine how GOLD reacts with RAGE to transmit a signal into the cell. The docking for Glide is to bind a flexible ligand to a rigid protein. MM-GBSA is a broadly used method for binding free energy estimation of a small molecule and receptor [49,50]. Molecular dynamics simulation, which is used to identify the precise binding model, is the basis of the MM-GBSA calculation, and the binding free energy is an important parameter of this calculation. To define an accurate binding model between GOLD and RAGE, MM-GBSA calculation was performed. We predicted the binding and structural association between GOLD and RAGE through the binding study of AGE and RAGE. The chemical and structural association of AGE and RAGE elicits a variety of intracellular responses and is associated with renal disorders. The interaction between AGEs and RAGE results in various outcomes, such as increased free radicals, increased oxidative stress, and decreased mitochondrial function, causing damage to the kidneys [51]. Therefore, the AGE–RAGE axis is an important factor when studying the effects of harmful AGEs on the kidneys. Our study showed that GOLD induced RAGE expression in kidney cells. This also confirms the importance of the structural bond between GOLD and RAGE in kidney damage. Therefore, we demonstrate that not only GOLD-induced RAGE production, but also the combination of AGEs and RAGE, are important and trigger a detrimental effect on the kidneys. These results show that regulation of the AGE–RAGE axis significantly affects mesangial cells. As a molecular mechanism involving glycation, AGEs bind to RAGE and accelerate processes such as fibrosis, apoptosis, and inflammation via subsequent activation of the AGE–RAGE axis [52]. Glyoxalase detoxifies AGE precursors such as glyoxal and methylglyoxal, thereby inhibiting the generation of AGEs and the activation of the AGE–RAGE axis [53]. The glyoxalase system catalyzes the precursors of AGEs using two thiol-dependent enzymes, GLO1/2 [54]. Therefore, the glyoxalase system essentially affects glycation stress signal removal induced by the AGE–RAGE axis.

AGEs induce ROS production by activating the signaling pathway via RAGE. Recent studies have suggested that the AGE–RAGE axis plays an essential role in ROS generation and gene activation [55,56]. Accumulated AGE also mediates several effects through RAGE, activating pathways that promote excessive production of ROS. Our research focuses on ROS production through the interaction of GOLD with RAGE. This process accelerates oxidative stress by reducing the activity of glyoxalase, which breaks down the precursors that produce AGEs. Eventually, the AGE–RAGE axis induces overproduction of intracellular ROS [56,57]. Furthermore, it worsens diseases and lesions through the activity of nicotinamide adenine dinucleotide phosphate (NADPH) oxidase and oxidative stress. During oxidative stress, AGEs induce excessive amounts of substances that damage cells, such as cytokines and ROS [58]. AGE is well known to be associated with ROS production and mitochondrial dysfunction in kidney-related diseases. In addition, the AGE–RAGE axis causes functional and morphological changes in mitochondria and an overproduction of ROS associated with kidney disease [56]. Therefore, we investigated the effect on mitochondrial dysfunction by a specific AGE, GOLD. Mitochondria are involved in many aspects, including the physiological and pathological roles of ROS production [57,59]. Thus, mitochondrial and ROS interactions suggest disease-related mechanisms. In this study, we examined the detrimental effects of ROS and the mechanisms involved in kidney damage and associated mitochondrial dysfunction. We identified a more accurate mechanism by identifying the link between RAGE-mediated ROS production and oxidative stress. It was confirmed that GOLD promotes RAGE expression and is involved in ROS production through oxidative stress. In this process, mitochondrial dysfunction was found to be caused by ROS production. Therefore, we emphasize that GOLD-induced mitochondrial dysfunction and excessive ROS production induce lesion exacerbation. It was also demonstrated that blocking ROS production and RAGE expression decreases the inflammatory response in mesangial cells.

Oxidative stress and inflammatory reactions are closely related to the development and progression of kidney disease. Oxidative stress that exacerbates kidney-related diseases is due to increased production of ROS and decreased antioxidant capacity [4,60]. The NRF2 transcription factor is well known, and oxidative stress is a key gene regulator. NRF2 activation regulates oxidative stress and inflammatory responses [19,22]. Based on the current observations, the production of ROS and changes in mitochondrial function due to the effect of GOLD suggest a change in NRF2 activity. NRF2 has been suggested to regulate inflammatory response-related factors, such as cytokines, and oxidative stress regulators depending on its transcription in mesangial cells [61].

## 5. Conclusions

This study demonstrated damage to the renal cells through GOLD–RAGE interaction in mesangial cells. GOLD induces excessive inflammatory reactions by binding to RAGE in mesangial cells. The detrimental action of GOLD on the inflammatory response is mediated by the NRF2/GLO1 signaling pathway in renal cells. In addition, ROS production caused by AGE–RAGE binding essentially worsens mesangial damage and is a critical factor in the disruption of mitochondrial function. In conclusion, we have confirmed that GOLD is naturally generated from the body environment. Furthermore, GOLD has been found to crucially influence renal cell damages. Therefore, this study shows the importance of reducing GOLD-induced damage in the body and that the damage caused by GOLD can be controlled.

## Figures and Tables

**Table 1:** Primer sequences for real-time PCR.

Gene	Forward Primer (5’ → 3’)	Reverse Primer (5’ → 3’)
RAGE	AAG CCC CTG GTG CCT AAT GAG	GAA TTC ATG GCA GAG CCA CAG CCG
NRF2	ATA TTC CCA GCC ACG TTG AG	AAC TTG CTC CAT GTC CTG CT
GLO1	GAA TTC ATG GCA GAG CCA CAG CCG	GAA TTC ATG GCA GAG CCA CAG CCG
GAPDH	TGC ATC CTG CAC CAC CAA	TCC ACG ATG CCA AAG TTG TC

## Data Availability

The data presented in this study are available in article.
